# A Role for Liquid-Ordered Plasma Membrane Nanodomains Coordinating the Unconventional Secretory Pathway of Fibroblast Growth Factor 2?

**DOI:** 10.3389/fcell.2022.864257

**Published:** 2022-04-01

**Authors:** Fabio Lolicato, Walter Nickel

**Affiliations:** Heidelberg University Biochemistry Center, Heidelberg, Germany

**Keywords:** fibroblast growth factor 2, unconventional protein secretion, protein self-translocation across membranes, lipidic membrane pores, phosphoinositides, heparan sulfates, glypican

## Abstract

Fibroblast growth factor 2 (FGF2) is a tumor cell survival factor that belongs to a subgroup of extracellular proteins lacking N-terminal signal peptides. Whereas this phenomenon was already recognized in the early 1990s, detailed insights into the molecular mechanisms underlying alternative pathways of protein secretion from eukaryotic cells were obtained only recently. Today, we know about a number of alternative secretory mechanisms, collectively termed unconventional protein secretion (UPS). FGF2 belongs to a subgroup of cargo proteins secreted by direct translocation across the plasma membrane. This feature has been classified as type I UPS and is shared with other unconventionally secreted proteins, such as HIV-Tat and Tau. FGF2 translocation across the membrane is initiated through sequential interactions with the Na,K-ATPase, Tec kinase, and phosphoinositide PI(4,5)P_2_ at the inner plasma membrane leaflet. Whereas the first two are auxiliary factors of this pathway, the interaction of FGF2 with PI(4,5)P_2_ triggers the core mechanism of FGF2 membrane translocation. It is based on a lipidic membrane pore that is formed by PI(4,5)P_2_-induced oligomerization of FGF2. Membrane-inserted FGF2 oligomers are recognized as translocation intermediates that are resolved at the outer plasma membrane leaflet by glypican-1, a heparan sulfate proteoglycan that captures and disassembles FGF2 oligomers on cell surfaces. Here, we discuss recent findings suggesting the molecular machinery mediating FGF2 membrane translocation to be highly organized in liquid-ordered plasma membrane nanodomains, the core process underlying this unusual pathway of protein secretion.

## Introduction

### The Unconventional Secretory Pathway of FGF2

As with many cargo proteins transported into the extracellular space by various types of unconventional protein secretion (UPS) pathways (Malhotra, 2013; Rabouille, 2017; Dimou and Nickel, 2018; Pallotta and Nickel, 2020), fibroblast growth factor 2 (FGF2) is a growth factor involved in fundamental biological processes, such as angiogenesis and wound healing (Beenken and Mohammadi, 2009). These functions of FGF2 are linked to its ability to form ternary signaling complexes with heparan sulfates and FGF high affinity receptors on cell surfaces (Plotnikov et al., 1999; Schlessinger et al., 2000). In addition to its role in development, FGF2 also plays key roles under pathophysiological conditions with both cancer cells and cells from their microenvironment producing vast amounts of FGF2 to trigger tumor-induced angiogenesis (Akl et al., 2016). Under certain circumstances, signaling cascades initiated by FGF2 can trigger immune escape mechanisms that lead to a block of apoptotic programs (Noh et al., 2014). For example, FGF2 causes chemoresistance in patients suffering from acute myeloid leukemia (Traer et al., 2016; Javidi-Sharifi et al., 2019). Despite the requirement of FGF2 to have access to the extracellular space to activate FGF receptors on cell surfaces, the analysis of its primary structure revealed the absence of a signal peptide for ER/Golgi-dependent protein secretion. However, even though major efforts were made, the proposed existence of alternative pathways of protein secretion (Muesch et al., 1990; Nickel, 2003) remained a hypothesis for decades as detailed insights into the molecular mechanism by which FGF2 and other UPS cargoes are transported into the extracellular space could be obtained only recently (Malhotra, 2013; Rabouille, 2017; Dimou and Nickel, 2018; Steringer and Nickel, 2018; Pallotta and Nickel, 2020).

**FIGURE 1:**
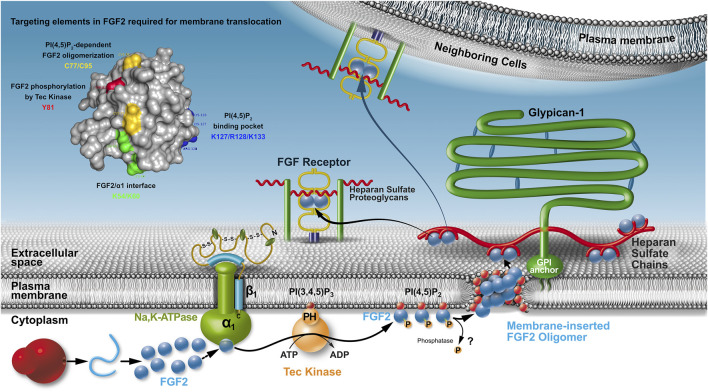
The unconventional secretory pathway of FGF2. Localization, molecular machinery, and mechanism as well as *cis* elements in FGF2 that are required for FGF2 translocation across the plasma membrane of eukaryotic cells. Whereas it is clear that the Na,K-ATPase are upstream of PI(4,5)P_2_, the order of sequential FGF2 interactions with the Na,K-ATPase and Tec kinase remains to be determined. It is also unclear whether FGF2 is secreted as a phosphorylated protein or whether a phosphatase removes this modification before FGF2 membrane translocation occurs.

All components of the molecular machinery mediating unconventional secretion of FGF2 have been found to be localized to the plasma membrane (Figure 1). These factors include the Na, K-ATPase (Zacherl et al., 2015; Legrand et al., 2020), Tec kinase containing a PH domain that binds to the phosphoinositide PI(3,4,5)P_3_ (Ebert et al., 2010; La Venuta et al., 2016; Steringer et al., 2012) as well as PI(4,5)P_2_, another phosphoinositide enriched in the inner leaflet of the plasma membrane (Nickel, 2011; Temmerman et al., 2008; Temmerman and Nickel, 2009). A cluster of amino acids with basic side chains (K127, R128, and K133; Figure 1) mediates PI(4,5)P_2_-dependent membrane recruitment of FGF2 (Temmerman et al., 2008; Steringer et al., 2017; Müller et al., 2015). This interaction initiates the core mechanism of FGF2 membrane translocation, a process that involves membrane insertion of FGF2 oligomers (Steringer and Nickel, 2018; Steringer et al., 2012; Steringer et al., 2017). Their biogenesis depends on the formation of intermolecular disulfide bridges (Dimou and Nickel, 2018; Steringer et al., 2017; Müller et al., 2015). As illustrated in Figure 1, the lipidic membrane pore that is induced by FGF2 oligomers is characterized by a toroidal architecture (Steringer et al., 2012; Müller et al., 2015; Steringer and Nickel, 2018). Several experimental observations support this view, such as the simultaneous membrane passage of fluorescent tracers and the transbilayer diffusion of membrane lipids that can be observed concomitant with PI(4,5)P_2_-dependent membrane insertion of FGF2 oligomers (Steringer et al., 2012; Steringer and Nickel, 2018). Furthermore, diacylglycerol, a cone-shaped lipid that interferes with PI(4,5)P_2_-induced positive membrane curvature inhibits membrane pore formation by FGF2 oligomers (Steringer et al., 2012; Steringer and Nickel, 2018). Finally, fusion proteins, such as FGF2-GFP form lipidic membrane pores with an increased pore size cutoff, a phenomenon that is reported previously for toroidal membrane pores (Gilbert et al., 2014). Therefore, PI(4,5)P_2_ plays multiple roles in FGF2 secretion with 1) mediating FGF2 membrane recruitment, 2) initiating FGF2 oligomerization, and 3) stabilizing positive membrane curvature to trigger the conversion of the lipid bilayer into a toroidal membrane pore with membrane-inserted FGF2 oligomers accommodated in its hydrophilic center (Dimou and Nickel, 2018; Steringer and Nickel, 2018). In this context, because FGF2 can attract multiple PI(4,5)P_2_ molecules, a strong local accumulation of this bilayer perturbing membrane lipid is proposed to compromise the integrity of the plasma membrane facilitating a membrane remodeling process converting the lipid bilayer into a toroidal membrane pore (Steringer et al., 2017; Dimou and Nickel, 2018; Pallotta and Nickel, 2020).

Based on the findings described above, membrane-inserted FGF2 oligomers are believed to be membrane translocation intermediates as part of an assembly/disassembly pathway that drives directional transport of FGF2 into the extracellular space (Dimou and Nickel, 2018; Steringer and Nickel, 2018). The final step of this process is mediated by cell surface heparan sulfate proteoglycans that capture FGF2 at the outer leaflet of the plasma membrane (Nickel, 2007; Nickel and Rabouille, 2009; Nickel and Seedorf, 2008; Zehe et al., 2006). Of note, compared with PI(4,5)P_2_, heparan sulfates exhibit an approximately hundredfold higher affinity toward FGF2 (Temmerman et al., 2008; Temmerman and Nickel, 2009; Steringer et al., 2017; Raman et al., 2003). Furthermore, the binding site in FGF2 for heparan sulfates and PI(4,5)P_2_ overlaps with some key residues, such as K133 being essential for both types of interactions (Temmerman et al., 2008; Temmerman and Nickel, 2009; Steringer et al., 2017). Consistently, interactions of FGF2 with heparan sulfate chains and PI(4,5)P_2_ are shown to be mutually exclusive (Steringer et al., 2017). These findings reveal a key aspect of the molecular mechanism of FGF2 membrane translocation providing a compelling explanation of how FGF2 assembles at the inner leaflet in a PI(4,5)P_2_-dependent manner into membrane-inserted oligomers that are captured and disassembled at the outer leaflet by cell surface heparan sulfate chains (Dimou and Nickel, 2018; Pallotta and Nickel, 2020; Rabouille, 2017). Thus, heparan sulfates mediate the last step of FGF2 membrane translocation with FGF2 being retained on cell surfaces (Figure 1). Following translocation into the extracellular space, FGF2 is capable of spreading to neighboring cells, probably mediated by direct exchange between heparan sulfate chains that are linked to proteoglycans on cell surfaces that are in close proximity (Zehe et al., 2006). Thus, from the biosynthesis of FGF2 on free ribosomes all the way to the cell surface, heparan sulfate proteoglycans exert multiple functions with 1) mediating the final step of FGF2 secretion (Nickel, 2007; Zehe et al., 2006), 2) protecting FGF2 on cell surfaces against degradation and denaturation (Nugent and Iozzo, 2000) and 3) mediating FGF2 signaling through ternary complexes in which FGF2, heparan sulfate chains, and FGF high-affinity receptors are engaged (Presta et al., 2005; Belov and Mohammadi, 2013; Ribatti et al., 2007). In conclusion, directional transport of FGF2 into the extracellular space depends on sequential interactions of FGF2 with PI(4,5)P_2_ at the inner leaflet and, bridged my membrane translocation intermediates, interactions with heparan sulfates on the cell surface (Figure 1). The proposed mechanism is further supported by previous studies demonstrating that FGF2 remains in a fully folded state during all steps of its unconventional secretory route (Backhaus et al., 2004; Torrado et al., 2009; Nickel, 2011), a phenomenon that reflects the requirement for the formation of defined oligomers during membrane insertion. These findings imply a quality control step that ensures secretion to be limited to FGF2 species that are biologically active (Torrado et al., 2009; Nickel, 2011).

Another aspect of quality control as part of the unconventional secretory pathway of FGF2 might be related to the role of the Na, K-ATPase in this process. Whereas its function may be restricted to building a landing platform as the first contact point of FGF2 at the inner plasma membrane leaflet (Legrand et al., 2020), it is also speculated that unconventional secretion of FGF2 could be linked to the regulation of the ATPase activity of the Na,K-ATPase (Pallotta and Nickel, 2020). Because FGF2 secretion involves the formation of a transient lipidic pore in the plasma membrane and FGF2 binds to a region in the cytoplasmic domain of the *α*-subunit of the Na,K-ATPase that contains its enzymatic activity (Legrand et al., 2020), it appears to be an intriguing hypothesis that FGF2 might upregulate the ATPase activity of this Na,K exchanger. This, in turn, might help to maintain the membrane potential under circumstances that trigger the formation of lipidic membrane pores during unconventional secretion of FGF2, a process that does not appear to compromise cell viability (Dimou and Nickel, 2018; Steringer and Nickel, 2018; Pallotta and Nickel, 2020).

The molecular mechanism illustrated in Figure 1 is also relevant for other unconventionally secreted proteins. For example, HIV-Tat and Tau are shown to directly translocate across plasma membranes to get access to the extracellular space. Like FGF2, these processes require physical interactions with PI(4,5)P_2_ at the inner leaflet and heparan sulfates at the outer leaflet of the plasma membrane (Rayne et al., 2010; Debaisieux et al., 2012; Zeitler et al., 2015; Agostini et al., 2017; Katsinelos et al., 2018; Merezhko et al., 2018). In addition, certain aspects of this process may also be relevant to the unconventional secretory mechanism of interleukin 1*β*, a process that, under certain physiological conditions, is based upon the formation of membrane pores that are triggered by PI(4,5)P_2_-dependent oligomerization of inflammasome-activated Gasdermin D (He et al., 2015; Martín-Sánchez et al., 2016; Brough et al., 2017; Evavold et al., 2017; Monteleone et al., 2018).

### Recent Evidence Suggesting Liquid-Ordered Nanodomains to Play a Role in Organizing the Molecular Machinery Mediating FGF2 Membrane Translocation

The molecular principles of the unconventional secretory pathway of FGF2 could be recapitulated recently by two complementary experimental approaches, the biochemical reconstitution of FGF2 membrane translocation using purified components and giant unilamellar vesicles (GUVs) (Steringer et al., 2017) as well as the real-time imaging of FGF2 membrane translocation in living cells using single molecule TIRF microscopy (Dimou et al., 2019). In these studies, the molecular mechanism of this process has been validated and is now established in the field as the best-characterized example for a UPS Type I pathway (Figure 1) (Dimou and Nickel, 2018; Pallotta and Nickel, 2020). However, a striking difference was observed when comparing these experimental systems concerning the kinetics by which FGF2 can physically traverse the membrane. Using purified components to reconstitute FGF2 membrane translocation, incubation times in the range of several tens of minutes were required to observe a substantial amount of GUVs into which FGF2 had translocated (Steringer et al., 2017). Similar observations are made in experimental systems reconstituting PI(4,5)P_2_-dependent FGF2 oligomerization and membrane insertion (Steringer et al., 2012). By contrast, the time interval required for FGF2 translocation from the inner to the outer leaflet of the plasma membrane in living cells was found to be in the range of 200 ms (Dimou et al., 2019). Thus, whereas the molecular requirements were found to be identical with PI(4,5)P_2_-dependent oligomerization and heparan sulfate-mediated capturing of FGF2 being essential for FGF2 membrane translocation in both experimental systems, a vast difference was observed with regard to kinetics. Most likely, several factors contribute to this phenomenon. For example, in the biochemical reconstitution system, PI(4,5)P_2_ is not present in an asymmetric distribution between the two leaflets that characterizes native plasma membranes. Further, whereas heparan sulfate chains on cell surfaces are contained in proteoglycans positioning them in a membrane-proximal manner, soluble heparin was added to the lumen of GUVs in reconstitution experiments (Steringer et al., 2017). Finally, auxiliary factors, such as the Na, K-ATPase, and Tec kinase, were absent in the *in vitro* reconstitution experiments. Whereas these factors probably affect the kinetics of FGF2 membrane translocation, they are unlikely to fully explain the vast difference of minutes versus milliseconds observed for this process when *in vitro* conditions (Steringer et al., 2012; Steringer et al., 2017) were compared with the authentic action observed in living cells (Dimou et al., 2019). What could be a compelling and testable explanation for the observed differences? An intriguing hypothesis would be the existence of nanodomains in native plasma membranes in which all components of the FGF2 secretion machinery are brought into proximity. As detailed below, recent studies indeed provide initial evidence for the structural organization of the FGF2 secretion machinery in specialized plasma membrane nanodomains.

### A Role for Liquid-Ordered Nanodomains as a Structural Platform of the FGF2 Secretion Machinery?

Several lines of evidence support the idea of a subpopulation of liquid-ordered membrane domains enriched in cholesterol and PI(4,5)P_2_ as platforms that host the machinery mediating FGF2 membrane translocation. First, in a recent study, cholesterol is demonstrated to be a critical factor affecting the ability of FGF2 to get recruited to membranes in a PI(4,5)P_2_-dependent manner with high binding strength and fast kinetics (Lolicato et al., 2021). The physiological relevance of this phenomenon could be confirmed in intact cells with increased levels of cholesterol resulting in higher efficiencies of FGF2 transport into the extracellular space. This study further provides insights into the molecular mechanism by which cholesterol affects both PI(4,5)P_2_-dependent recruitment and membrane translocation of FGF2 using molecular dynamics simulations. An increase of cholesterol at the expense of phosphatidylcholine, a scenario that mimics the changes in membrane lipid compositions when plasma membranes are compared with the endoplasmic reticulum, caused two phenomena. First, the visibility of the head group of PI(4,5)P_2_ was found to be increased, facilitating FGF2 binding to lipid bilayers. Second, in the presence of increased levels of cholesterol, PI(4,5)P_2_ was found to cluster forming trimers and tetramers. This, in turn, causes an increase in avidity, explaining faster binding kinetics and an enhanced binding strength of FGF2 toward PI(4,5)P_2_ (Lolicato et al., 2021). The observed effects of cholesterol could also be directly relevant for the subsequent oligomerization of FGF2. Using molecular dynamics simulations, it was found that cholesterol-containing membranes stabilize an orientation of PI(4,5)P_2_ that promotes the formation of disulfide-linked dimers of FGF2 (Steringer et al., 2017). Under these conditions, in addition to the defined high-affinity PI(4,5)P_2_ binding site in FGF2, additional PI(4,5)P_2_ molecules were found to bind to FGF2 at other sites. The role of PI(4,5)P_2_ in this process is a highly specific one as artificial membrane anchors, such as a Ni-NTA lipid along with a His-tagged version of FGF2, were found incapable of forming functional FGF2 oligomers that form lipidic membrane pores as transient intermediates in unconventional secretion of FGF2 (Steringer et al., 2012; Steringer et al., 2017). Beyond the abovementioned studies on FGF2, an enrichment of PI(4,5)P_2_ in liquid-ordered domains organized by cholesterol has indeed been recognized in other studies as well (Myeong et al., 2021; Wen et al., 2021).

Another recent study added further support to the idea that FGF2 membrane translocation occurs in cholesterol-enriched plasma membrane nanodomains characterized by a liquid-ordered state. In a BioID screen probing for proteins that are in proximity of FGF2 at any time point of its lifetime in cells, a specific type of heparan sulfate proteoglycan has been identified as the key driver of FGF2 secretion, Glypican-1 (GPC1) (Sparn et al., 2021). Whereas a knockout of GPC-1 was found to cause a substantial decrease in FGF2 secretion efficiencies, overexpression of GPC1 did not only rescue to wild-type levels but rather significantly increased FGF2 secretion rates. Furthermore, biochemical analyses revealed that the heparan sulfate chains of GPC1 contain high-affinity sites for FGF2 that are less present in other heparan sulfate proteoglycans, including members of the glypican and syndecan families. Of note, like all glypicans, GPC1 is associated with the outer leaflet of the plasma membrane *via* a GPI anchor (Filmus et al., 2008). Like other membrane proteins with GPI anchors, GPC1 is known to partition into liquid-ordered plasma membrane domains on cell surfaces. In addition, GPC1 contains a large N-terminal domain that builds a lid-like structure with a length of about 10 nm on top of the membrane. The N-terminal lid domain is connected to the GPI anchor *via* a linker to which three heparan sulfate chains are attached. They are oriented in a highly membrane-proximal manner with the distance between them and the membrane surface being just about 3 nm. These observations imply that the prominent role of GPC1 in unconventional secretion of FGF2 is promoted by its unique structure that appears to form a microenvironment between the GPC1 lid and the membrane surface in which high-affinity binding sites for FGF2 are arranged in a membrane-proximal and highly concentrated manner.

## Discussion

Whereas the principal molecular components and mechanisms of the molecular machinery mediating unconventional secretion of FGF2 have been identified, its spatiotemporal organization at the plasma membrane is unknown. With recent findings demonstrating a role for cholesterol promoting efficient binding of FGF2 to PI(4,5)P_2_ concomitant with increased FGF2 secretion rates, the identification of PI(4,5)P_2_ clusters in liquid-ordered domains and the identification of GPC1, a GPI-anchored heparan sulfate proteoglycan that partitions into liquid-ordered domains, being the key driver of the unconventional secretory pathway of FGF2, we propose all components of this pathway to reside in plasma membrane nanodomains in a highly organized manner. It will be of great interest to challenge this hypothesis further, in particular with regard to the Na, K-ATPase that is the initial contact point for FGF2 at the inner plasma membrane leaflet. It will be an important future goal to reconstitute this type of nanodomain with purified components to uncover the mechanisms underlying the vast kinetic differences that have been found between *in vitro* reconstitution experiments and the authentic action observed in living cells. A comprehensive understanding of this pathway will not only solve a long-standing problem in molecular cell biology, but will also pave the way for the development of new inhibitors that have great potential for cancer therapy, for example, in fighting chemoresistances that are caused by FGF2 in acute myeloid leukemia.
